# GDF-11 Protects the Traumatically Injured Spinal Cord by Suppressing Pyroptosis and Necroptosis via TFE3-Mediated Autophagy Augmentation

**DOI:** 10.1155/2021/8186877

**Published:** 2021-10-19

**Authors:** Yu Xu, Xinli Hu, Feida Li, Haojie Zhang, Junsheng Lou, Xingyu Wang, Hui Wang, Lingyan Yin, Wenfei Ni, Jianzhong Kong, Xiangyang Wang, Yao Li, Kailiang Zhou, Hui Xu

**Affiliations:** ^1^Department of Orthopaedics, The Second Affiliated Hospital and Yuying Children's Hospital of Wenzhou Medical University, Wenzhou 325027, China; ^2^Zhejiang Provincial Key Laboratory of Orthopaedics, Wenzhou 325027, China; ^3^The Second Clinical Medical College of Wenzhou Medical University, Wenzhou 325027, China

## Abstract

Spinal cord injury (SCI) refers to a major worldwide cause of accidental death and disability. However, the complexity of the pathophysiological mechanism can result in less-effective clinical treatment. Growth differentiation factor 11 (GDF-11), an antiageing factor, was reported to affect the development of neurogenesis and exert a neuroprotective effect after cerebral ischaemic injury. The present work is aimed at investigating the influence of GDF-11 on functional recovery following SCI, in addition to the potential mechanisms involved. We employed a mouse model of spinal cord contusion injury and assessed functional outcomes via the Basso Mouse Scale and footprint analysis following SCI. Using western blot assays and immunofluorescence, we analysed the levels of pyroptosis, autophagy, necroptosis, and molecules related to the AMPK-TRPML1-calcineurin signalling pathway. The results showed that GDF-11 noticeably optimized function-related recovery, increased autophagy, inhibited pyroptosis, and alleviated necroptosis following SCI. Furthermore, the conducive influences exerted by GDF-11 were reversed with the application of 3-methyladenine (3MA), an autophagy suppressor, indicating that autophagy critically impacted the therapeutically related benefits of GDF-11 on recovery after SCI. In the mechanistic study described herein, GDF-11 stimulated autophagy improvement and subsequently inhibited pyroptosis and necroptosis, which were suggested to be mediated by TFE3; this effect resulted from the activity of TFE3 through the AMPK-TRPML1-calcineurin signalling cascade. Together, GDF-11 protects the injured spinal cord by suppressing pyroptosis and necroptosis via TFE3-mediated autophagy augmentation and is a potential agent for SCI therapy.

## 1. Introduction

Spinal cord injury (SCI) refers to a destructive disease that causes serious neurological and motor dysfunctions when the structure is injured [1]. Clinically, a few SCI therapies, including surgeries, increased blood pressure, and methylprednisolone administration, are able to decompress and stabilize injuries, preventing secondary complications and managing symptoms [2–4]. The SCI pathology is complicated and operationally falls into two states: primary injury triggered by mechanical damage, which includes demyelination and necrosis of axons and neurons, and secondary injury initiated by a variety of pathophysiologies, including autophagy, apoptosis, oxidative stress, inflammation, and other factors, which primarily aggravate neurological dysfunction and could be reversible and regulated [5–7]. Thus, secondary injury prevention and intervention are considered promising treatments for SCI [8]. Among the mentioned pathophysiologic events in secondary injury, cell death and inflammation are considered to be two critical targets for SCI treatment [9, 10].

Pyroptosis, a proinflammation-related programmed cell death pathway, markedly impacts neuroinflammation, a critical element that drives secondary injury post-SCI and is induced through inflammasome activation [11, 12]. The inflammasome refers to a complex of multimeric proteins that include a cytosolic sensor (e.g., NLRP1, NLRP2, NLRP3, etc.), an adaptor protein (ASC), and an effector caspase (caspase-1) [13, 14]. By stimulating the cytoplasmic inflammasome complex, caspase-1/4/5/11 are activated and the gasdermin- (GSDMD-) *N* domain is translocated to the cell membrane, thereby inducing pore formation and causing pyroptosis [15–17]. Necroptosis, another type of proinflammation-related programmed cell death, is activated by the binding of tumour necrosis factor-*α* (TNF-*α*) to tumour necrosis factor receptor 1 (TNFR1) and is involved in the intracellular signalling cascade involving receptor-interacting protein kinase 1/3 (RIPK1/3) and mixed lineage kinase domain-like (MLKL) protein [18]. Moreover, it was demonstrated that caspase-8 suppresses the occurrence of necroptosis [19]. An increasing number of studies have shown that necroptosis and pyroptosis cause numerous neurological diseases, including neurodegenerative disorders, ischaemic brain injury, and psychiatric diseases [20, 21]. Additionally, necroptosis and pyroptosis aggravate neuronal and glial cell death after SCI [22, 23]. Therefore, targeting necroptosis and pyroptosis is promising as a potential therapy for SCI.

Macroautophagy (hereafter named autophagy) is a dynamic regulatory mechanism that maintains the stability of intracellular environments and degrades long-lived proteins and damaged organelles in a selective manner [24, 25]. According to accumulating evidence, autophagy has a critical effect after neurological diseases by regulating neural cell death [26, 27]. According to SCI pathogenesis, lysosomal injury and dysfunction can trigger defects within autophagy fluxes and messy environments, triggering an inflammatory response [28]. Subsequently, the activation of NLRP3 inflammasomes facilitates the cleavage of pro-IL-1*β*/18 and cleaves GSDMD into 2 parts, ultimately initiating pyroptosis [29–32]. Moreover, it has been found that lysosomal damage decreases autophagy flux, leading to rapid increases in the necroptosis activators RIPK1, RIPK3, and MLKL in neurons [33]. Moreover, recent studies have also demonstrated that autophagy can suppress pyroptosis and necroptosis by degrading key activators [34–36]. Thus, we hypothesized that autophagy may modulate the death of neural cells for neuroprotection by suppressing pyroptosis and necroptosis and it may improve functional recovery after SCI. Accordingly, active drugs capable of inhibiting pyroptosis and necroptosis through the activation of autophagy should be identified.

Growth differentiation factor 11 (GDF-11) pertains to the transforming growth factor *β* (TGF-*β*) superfamily, members of which impact numerous processes, such as histogenesis, embryonic development, cancer, and metabolic disorders [37, 38]. A recent study has indicated that GDF-11 exerts its neuroprotective effect in cerebral ischaemic injury to reduce neuronal apoptosis [39]. GDF-11 treatment also improves functional outcomes and stimulates neurogenesis and angiogenesis in mice by activating autophagy in ischaemic stroke [40]. However, the effects exerted by GDF-11 in the treatment of SCI have never been investigated. Furthermore, whether GDF-11 inhibits pyroptosis and necroptosis by activating autophagy remains unknown. Therefore, we assessed the effect of GDF-11 on autophagy, pyroptosis, and necroptosis and assessed its effects on the functional recovery following SCI using a mouse model of SCI injected with GDF-11.

## 2. Material and Method

### 2.1. Animals and Ethics Statement

Healthy adult C57BL/6 mice (female, average weight 20–30 g) originated from Wenzhou Medical University's Experimental Animal Center (license no. SCXK [ZJ] 2015-0001), Zhejiang province, China. All animals were housed under standard conditions (temperature: 21–25°C, 12 h light/dark cycle, humidity: 50–60%) with free access to water and food. The experimental procedure related to animals followed the Guide for the Care and Use of Laboratory Animals of the China National Institutes of Health, as accepted by the Animal Care and Use Committee of Wenzhou Medical University (wydw 2017-0022).

### 2.2. Antibodies and Reagents

The chemicals listed below were used herein: GDF-11 was acquired from PeproTech (Rocky Hill, the United States of America; Cat# 120-11). Solarbio Science & Technology (Beijing, China) provided the pentobarbital sodium, diaminobenzidine (DAB) developer, Masson staining tool, and haematoxylin and eosin (HE) staining tools. Sigma-Aldrich (St. Louis, MO, the United States of America; Cat# M9281) supplied the 3-methyladenine (3MA). MedChemExpress (Monmouth Junction, NJ, the United States of America; Cat# HY-13418A) provided the dorsomorphin (Compound C, C24H25N5O; purity ≥98.14%). Shanghai Genechem Co. Ltd. (Shanghai, China) developed the adeno-associated virus transcription factor E3 (AAV-TFE3) shRNA (serotype 9, with no fluorescent reporter gene). Thermo Fisher Scientific (Rockford, IL, United States of America) provided the Cytoplasmic Extraction Reagent and NE-PER™ Nuclear and BCA tools. Cell Signaling Technology (Beverly, MA, United States of America; Cat# 3738, Cat# 15101, Cat# 5832, Cat# 2535, Cat# 2983, and Cat# 5536) supplied the primary antibodies against Beclin1, NLRP3, AMPK, p-AMPK, p-mTOR, and mTOR. Proteintech Cohort (Chicago, IL, United States of America; Cat# 12452-1, Cat# 21327-1, Cat# 22915-1, Cat# 17168-1, and Cat# 10494-1) provided the VPS34, CTSD, CASP1, histone H3, and GAPDH antibodies. Abcam (Cambridge, UK; Cat# ab106393, Cat# ab62344, Cat# ab180799, Cat# ab207323, Cat# ab52761, Cat# ab52636, Cat# ab183830, Cat# ab104224, Cat# ab272608, Cat# ab56416, Cat# ab150077, and Cat# ab150115) provided the goat anti-mouse IgG H&L (Alexa Fluor® 647), RIPK1, RIPK3, ASC, IL-18, calcineurin, synaptophysin (SYN), microtubule-associated protein-2 (MAP2), NeuN, TRPML1/MG-2, p62/SQSTM1, and goat anti-rabbit IgG H&L (Alexa Fluor® 488) antibodies. The primary antibody against TFE3 was purchased from Sigma-Aldrich Chemical Company (Milwaukee, WI, United States of America; Cat# HPA023881). LC3B was purchased from Novus Biologicals (Littleton, CO, United States of America; Cat# NB600-1384). IL-1*β* was purchased from ABclonal Technology (Cambridge, MA, United States of America; Cat# A1112). MLKL, GSDMD, and NLRP1 were from Affinity Biosciences (OH, United States of America; Cat# DF7412, Cat# AF4013, and Cat#DF13187). Santa Cruz Biotechnology (Dallas, TX, the United States of America) provided the horseradish peroxidase- (HRP-) conjugated IgG secondary antibody. Boyun Biotechnology (Nanjing, the People's Republic of China) provided the fluorescein isothiocyanate- (FITC-) conjugated IgG secondary antibody. Furthermore, Beyotime Biotechnology (Jiangsu, the People's Republic of China) supplied the 4′,6-diamidino-2-phenylindole (DAPI) solution.

### 2.3. Animal Model of SCI

In advance of the procedures, anaesthesia was administered to animals by intraperitoneal injection with 1% (*w*/*v*) pentobarbital sodium (50 mg/kg). Next, a standard laminectomy was performed in the T9-T10 vertebra to expose a dura circle. Subsequently, a weight drop injury model was adopted to trigger spinal cord contusion injury following a previous description [41]. Briefly, we dropped a bar (10 g in weight and 1.2 mm in diameter) from 15 mm onto the exposed spinal cord to induce moderate SCI contusion while keeping the dura intact. After the injury, the layers of the skin, fascia, and muscle were closed with 4–0 nonabsorbable silk sutures. Mice in the sham group underwent the same operation as mentioned above, with no injury caused by a weight drop. Following the procedure, the mice were artificially urinated three times per day.

### 2.4. Adeno-Associated Virus (AAV) Vector Packaging

The Shanghai Genechem Company developed the AAV-TFE3-shRNA. The shRNA sequence of TFE3-stimulated protein kinase was constructed, cloned, and then processed into the pAV-U6-shRNA-CMV-EGFP plasmid, and pAV-U6-shRNA(TFE3)-CMV-EGFP was obtained. AAV-293 cells were transfected to produce AAV9-U6-shRNA(TFE3)-CMVEGFP based on the AAV Rep/Cap expression plasmid, adenovirus helper plasmid (Ad helper), and pAV-U6-shRNA(TFE3)-CMV-EGFP. Likewise, AAV9-U6-shRNA(scramble)-CMV-EGFP was used as a scramble control. Viral particles were purified using the iodixanol gradient approach. Using quantitative PCR, we detected the titres of AAV9-U6-shRNA(TFE3)-CMV-EGFP and AAV9-U6-shRNA(scramble)-CMV-EGFP, i.e., 4.82 × 10^12^ and 6.56 × 10^12^ genomic copies per ml, respectively.

### 2.5. Drug and AAV Vector Administration

We separated 114 mice in a random manner into seven groups: sham (*n* = 18), SCI (*n* = 18), GDF-11 (*n* = 18), GDF − 11 + 3MA (*n* = 18), GDF − 11 + scrambled shRNA control (*n* = 18), GDF − 11 + TFE3 shRNA (*n* = 18), and GDF − 11 + CC (*n* = 6). The GDF-11 group was treated with GDF-11 (100 ng/kg/day) via daily intraperitoneal injection for 3 days after SCI [42]. An equal volume of saline was given to the sham and SCI groups. Daily intraperitoneal injection of 3-methyladenine (3MA, 15 mg/kg) and dorsomorphin (compound C, 1.5 mg/kg) was performed 30 min prior to GDF-11 administration for 3 days. The GDF − 11 + scrambled shRNA control and GDF − 11 + TFE3 shRNA groups received a 100 *μ*l intravenous injection of the viral vectors in PBS with 1 × 10^10^ packaged genomic particles 14 days before SCI. After 14 days, the GDF − 11 + scrambled shRNA control and GDF − 11 + TFE3 shRNA groups received the same treatment as the GDF-11 group. The animals were killed by overdosing them with pentobarbital sodium, and histological samples were acquired for corresponding experiments on days 3 and 28.

### 2.6. Functional Behavioural Assessment

We administered the Basso Mouse Scale (BMS) to measure locomotion on days 0, 1, 3, 7, 14, 21, and 28 following SCI to evaluate functional recovery [43]. BMS scores ranged from 0 to 9, with 0 indicating normal motor function and 9 indicating overall paralysis. We carried out a footprint investigation at 28 days after surgery. Hind limbs (red) and mouse forelimbs (blue) were stained using dyes of various colours [44]. Two independent testers without any knowledge of the experimental conditions measured the results.

### 2.7. Tissue Slide Preparation for HE and Masson Staining

On day 28 after surgery, the mice underwent reanaesthetization using 2% (*w*/*v*) pentobarbital sodium, perfusion using normal saline, and introduction of 4% (*w*/*v*) paraformaldehyde in phosphate-buffered saline. Next, we split the overall parts (10 mm long, epicentre in the centre) and fixed the mentioned parts in 4% (*w*/*v*) paraformaldehyde over a period of 24 h. Next, we developed the respective longitudinal paraffin sections after embedding the samples in paraffin. Using a microtome, 4 *μ*m sections were cut and mounted onto slides coated with poly-L-lysine to carry out HE staining-based histopathological tests following reported descriptions [45, 46]. For Masson staining, we employed 10% trichloroacetic acid and 10% potassium dichromate for mordant longitudinal sections. Haematoxylin was used to stain nuclei. Next, using ethanol and hydrochloric acid, the slides were differentiated, returned to blue with reduced ammonia, and stained with Masson solution. Staining was performed as described previously [47]. Finally, a light microscope (Olympus, Tokyo, Japan) was used to acquire images.

### 2.8. Western Blot (WB)Analysis

Mice were euthanized on day 3 under SCI, and the spinal cord parts from mice (1.5 cm; covering the injury epicentre) were dissected and stored at −80°C prior to WB. Partial samples were processed by extracting proteins with lysis buffer. Other samples were processed for extraction of cytoplasmic and nuclear proteins with Cytoplasmic Extraction Reagent and NE-PER™ Nuclear. We employed the protein extraction reagents to purify overall proteins from the spinal cord specimens. BCA assays were used for protein quantification. We performed 12% (*w*/*v*) gel electrophoresis to separate equal amounts of protein (60 *μ*g); the samples were then transferred to polyvinylidene fluoride membranes (Roche Applied Science, Indianapolis, IN, the United States of America), which were blocked in 5% (*w*/*v*) skimmed milk and probed with the following antibodies overnight at 4°C: ASC (1 : 1000), NLRP1 (1 : 1000), NLRP3 (1 : 1000), IL-18 (1 : 1000), IL-1*β* (1 : 1000), GSDMD (1 : 1000), CASP1 (1 : 1000), Beclin1 (1 : 1000), SQSTM1/p62 (1 : 1000) LC3B (1 : 1000), VPS34 (1 : 1000), CTSD (1 : 1000), RIPK1 (1 : 1000), RIPK3 (1 : 1000), MLKL (1 : 1000), CASP8 (1 : 1000), p-mTOR (1 : 1000), mTOR (1 : 1000), p-AMPK (1 : 1000), AMPK (1 : 1000), GAPDH (1 : 1000), CaPML1 (1 : 1000), histone (1 : 1000), p-AMPK (1 : 1000), and AMPK (1 : 1 : 1000). The membranes were subsequently incubated with HRP-conjugated IgG secondary antibodies at an ambient temperature for 2 h. Using a ChemiDoc™ XRS+ Imaging System (Bio-Rad) based on an ECL immune-detection tool, band signals were visualized and investigated.

### 2.9. Immunofluorescence (IF) Staining

On day 3 after SCI, spinal cord specimens from mice were dissected and collected for IF staining. We performed IF staining on the tissue side according to the rostral spinal cord (1 mm long, 4 mm from the epicentre) following a previous description [48]. We deparaffinized, rehydrated, washed, and then treated the sections with 10.2 mM sodium citrate buffer for 20 min at 95°C. Subsequently, we permeabilized the sections with 0.1% (*v*/*v*) PBS-Triton X-100 (10 min). Next, we blocked the sections with 10% (*v*/*v*) bovine serum albumin in PBS (1 h). The slides were then incubated overnight at 4°C with antibodies against Caspase-1 (1 : 200)/NeuN (1 : 400), GSDMD (1 : 150)/NeuN (1 : 400), LC3 (1 : 200)/NeuN (1 : 400), p62 (1 : 200)/NeuN (1 : 400), RIPK1 (1 : 200)/NeuN (1 : 400), RIPK3 (1 : 200)/NeuN (1 : 400), SYN (1 : 200)/NeuN (1 : 400), and MAP2 (1 : 200). Next, we washed the sections for 10 min at an ambient temperature 3 times and incubated them at an ambient temperature for 1 h with FITC-conjugated secondary antibody. Finally, we captured and evaluated images with a fluorescence microscope (Olympus, Tokyo, Japan) within six fields taken in a random manner in three random sections pertaining to the respective sample.

### 2.10. Statistical Analysis

We completed all statistical investigations using SPSS ver. 19 software (SPSS, Chicago, IL) and adopted a double-blind approach during the analysis process. Values are expressed as the mean ± standard error of the mean (SEM). To control for unwanted sources of variation, data normalization was performed in this study. Analysis of variance (ANOVA) based on the least significance difference (LSD) (equal variances assumed) post hoc investigation or Dunnett's T3 (equal variances without any assumption) approach was conducted to assess notable distinctions of two groups among three or four groups. We employed an independent sample *t*-test to compare two independent groups. The *p* values less than 0.05 indicated statistical significance.

## 3. Results

### 3.1. GDF-11 Facilitates Functional Recovery after SCI

**Figure 1:**
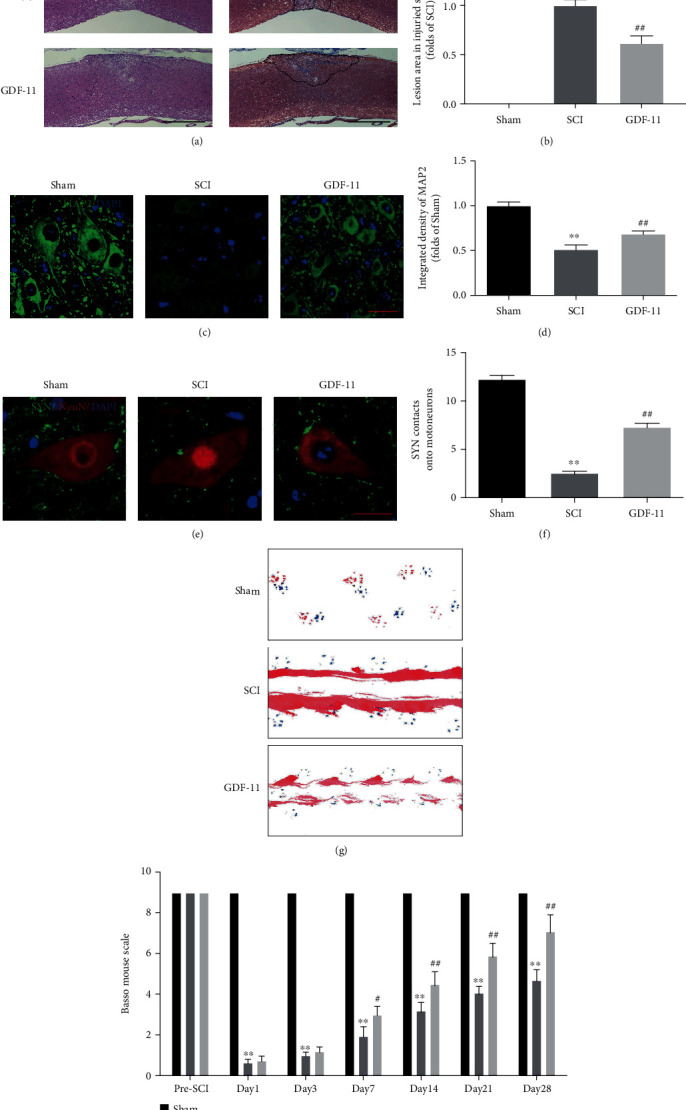
GDF-11 facilitates functional recovery following spinal cord injury. (a) Longitudinal spinal cord sections in the indicated groups on day 28 following SCI were analysed on the basis of Masson staining and HE staining (scale bar = 1000 *μ*m). (b) Quantitative investigations of Masson-positive lesions within the spinal cord of the respective groups. (c) Photographs (×30) of spinal cord sections in the respective groups stained with an antibody against MAP2 (scale bar = 25 *μ*m). (d) MAP2 optical density within a spinal cord subjected to injury on day 28. (e) Photographs (×150) of spinal cord sections subjected to injury (T11-T12) and stained on day 28 with an antibody against SYN/NeuN (scale bar = 5 *μ*m). (f) Relevant quantitative results for neuron-contacting synapse amounts. (g) Photographs of mouse footprints on day 28 following spinal cord injury. (h) Basso mouse scale (BMS) scores in the indicated groups and time points. Data are expressed as the mean ± SEM, *n* = 6 per group. ^∗∗^*p* < 0.01 vs. the sham group. ^#^*p* < 0.05 and ^##^*p* < 0.01 vs. the SCI group.

HE and Masson staining, IF staining, footprint analysis, and BMS scores were used to evaluate motor function following SCI. The lesion area in the injured spinal cord was assessed by HE and Masson staining, which showed a glial scar area with marked expansion (*p* < 0.01) in the SCI group compared with the sham group. Additionally, IF staining revealed reduced MAP2 expression (*p* < 0.01) and a smaller number of SYN-positive synapses on neurons (*p* < 0.01) in the SCI group than in the sham group. With GDF-11 treatment, the animals had fewer glial scars, greater neuronal MAP2 expression levels, and a greater number of SYN-positive synapses on neurons than the SCI group without any treatment (*p* < 0.01 for all; Figures 1(a)–1(f)). Next, in the footprint investigation, the GDF-11 group outperformed the SCI group in terms of functional recovery on day 28 after injury (Figure 1(g)). In the sham group, the BMS score markedly exceeded that in the SCI group on days 1, 3, 7, 14, 21, and 28 following the procedure (*p* < 0.01 for all). An insignificant difference was reported for the BMS score between the SCI and GDF-11 groups on days 1 and 3. Likewise, the GDF-11 group achieved greater BMS scores on days 7, 14, 21, and 28 following the procedure compared with the SCI group (*p* = 0.02, <0.01, <0.01, and <0.01, respectively; Figure 1(h)). Overall, the mentioned outcomes confirmed that GDF-11 facilitated functional recovery following SCI.

### 3.2. GDF-11 Attenuates Pyroptosis after SCI

**Figure 2:**
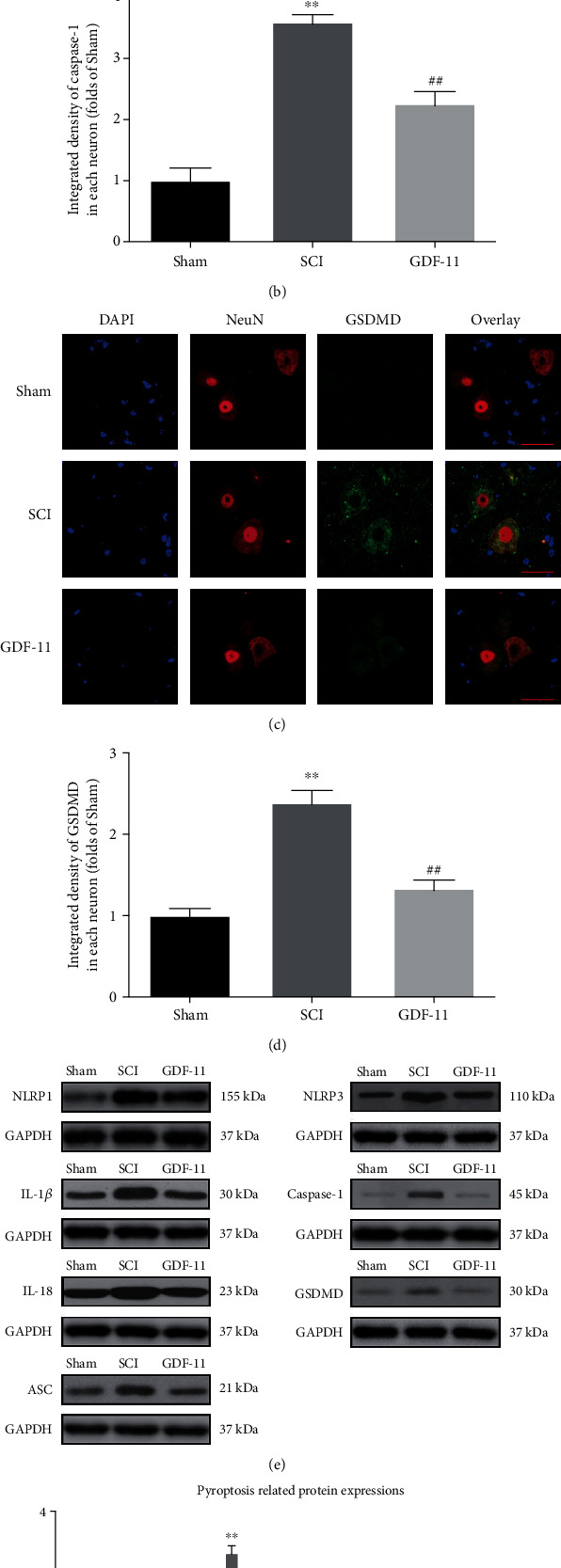
GDF-11 attenuates pyroptosis following spinal cord injury. (a) Immunofluorescence staining for Caspase-1 and NeuN colocalization in the spinal cords of the GDF-11, SCI, and sham groups (scale bar = 25 *μ*m). (b) The quantitative mean optical density of Caspase-1 in neurons of spinal cord lesions. (c) Immunofluorescence staining for GSDMD and NeuN colocalization in the spinal cords of the GDF-11, SCI, and sham groups (scale bar = 25 *μ*m). (d) The quantitative average optical density of GSDMD within neurons of spinal cord lesions. (e) Western blot assay for IL-18, IL-1*β*, GSDMD, Caspase-1, ASC, NLRP3, and NLRP1 expression levels in the three groups. Gels were subjected to identical experimental conditions, with cropped blots presented. (f) Optical densities of the IL-18, IL-1*β*, GSDMD, Caspase-1, ASC, NLRP3, and NLRP1 expression levels were quantified and investigated in the respective groups. Data are expressed as the mean ± SEM, *n* = 6 per group. ^∗∗^*p* < 0.01 vs. the sham group. ^#^*p* < 0.05 and ^##^*p* < 0.01 vs. the SCI group.

IL-18, IL-1*β*, NLRP1, NLRP3, Caspase-1, GSDMD, and ASC were assessed within the spinal cord following SCI to evaluate the pyroptotic activity in the GDF-11, SCI, and sham groups. As revealed by IF staining, compared with that in the sham group, GSDMD and Caspase-1 achieved marked increases in density in neurons within spinal cord lesions in the SCI group (*p* < 0.01 for both), whereas GDF-11 showed a downregulation of GSDMD and Caspase-1 density in comparison to that in the SCI group (*p* < 0.01 for both), as shown in Figures 2(a)–2(d). WB analysis of IL-18-, IL-1*β*-, NLRP1-, NLRP3-, Caspase-1-, GSDMD-, and ASC-expressing states was performed (Figure 2(e)). As revealed by the results, in contrast to the sham group, the SCI group achieved higher optical density (OD) values in terms of IL-18, IL-1*β*, GSDMD, Caspase-1, ASC, NLRP3, and NLRP1 (*p* < 0.01 for all) and GDF-11 showed decreased OD values for the mentioned markers compared with the SCI group (*p* = 0.02, =0.02, <0.01, <0.01, =0.04, <0.01, and <0.01, separately; Figure 2(f)). These results indicated that GDF-11 could reduce pyroptosis-associated markers, demonstrating suppression of pyroptosis after SCI.

### 3.3. GDF-11 Inhibits Necroptosis after SCI

**Figure 3:**
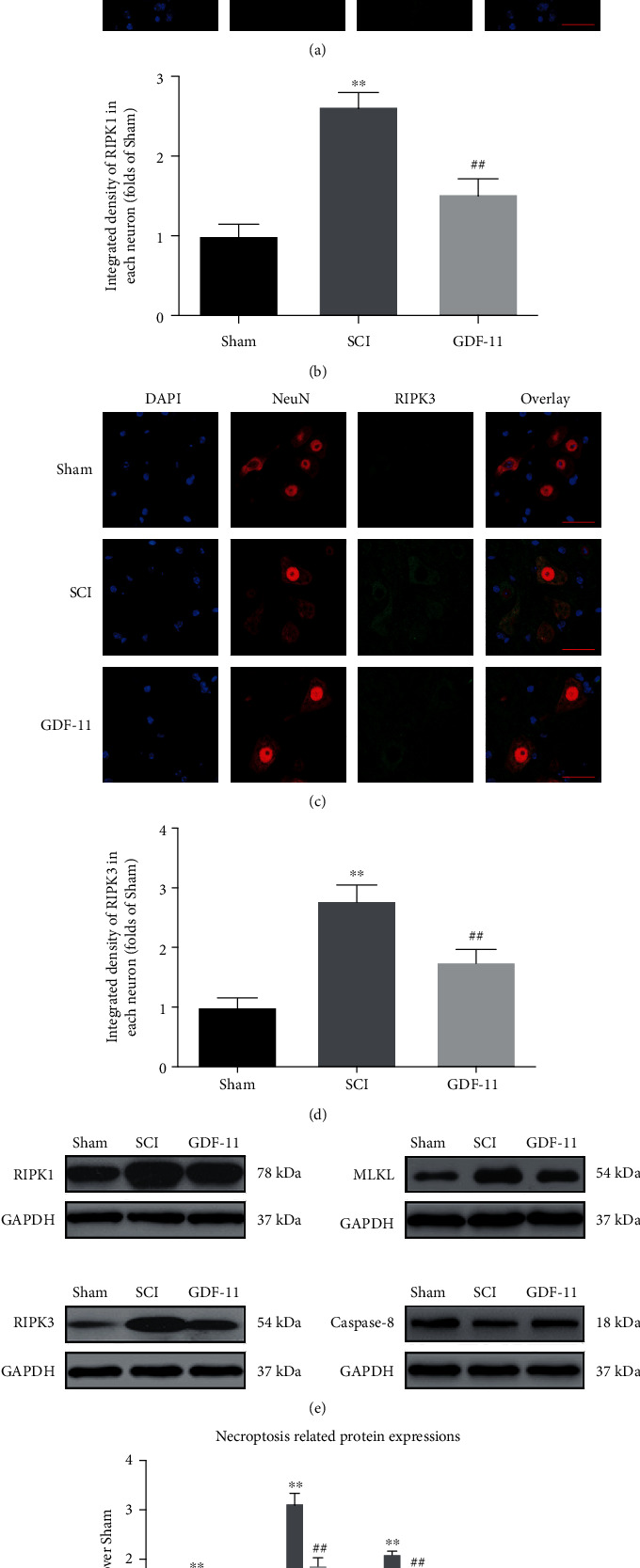
GDF-11 inhibits necroptosis following spinal cord injury. (a) Immunofluorescence staining for RIPK1 and NeuN colocalization in the spinal cords of the GDF-11, SCI, and sham groups (scale bar = 25 *μ*m). (b) Quantification of the optical density of RIPK1 in neurons of spinal cord lesions. (c) Immunofluorescence staining for RIPK3 and NeuN colocalization in spinal cords belonging to the GDF-11, SCI, and sham groups (scale bar = 25 *μ*m). (d) Quantification of the optical density of RIPK3 in neurons of spinal cord lesions. (e) Western blot assay for Caspase-8, MLKL, RIPK3, and RIPK1 expression levels in the GDF-11, SCI, and sham groups. Gels were subjected to identical experimental conditions, with cropped blots presented. (f) Optical densities of Caspase-8, MLKL, RIPK3, and RIPK1 expression levels were quantified and investigated in the respective groups. Data are expressed as the mean ± SEM, *n* = 6 per group. ^∗∗^*p* < 0.01 vs. the sham group. ^#^*p* < 0.05 and ^##^*p* < 0.01 vs. the SCI group.

We examined the states of necroptosis through WB and IF. As revealed by IF staining, relative to that in the sham group, the RIPK3 and RIPK1 density in neurons increased markedly within the spinal cord lesions in the SCI group (*p* < 0.01 for both), whereas GDF-11 showed a decreased density of RIPK3- and RIPK1-positive neurons in comparison to that in the SCI group (*p* < 0.01 for both), as shown in Figures 3(a)–3(d). Moreover, the expression levels of Caspase-8, MLKL, RIPK3, and RIPK1 were analysed by WB (Figure 3(e)). In comparison to those within the sham group, RIPK1, RIPK3, and MLKL showed greater OD values within the SCI group, while those for Caspase-8 were lower (*p* < 0.01 for all). In comparison to those in the SCI group, the expression levels of MLKL, RIPK3, and RIPK1 were markedly reduced in the GDF-11 group, while the expression of Caspase-8 was increased (*p* = 0.04, <0.01, <0.01, and =0.02; Figure 2(f)). Overall, these results indicated that the restorative effect of GDF-11 after SCI was partly due to the suppression of necroptosis.

### 3.4. GDF-11 Enhances Autophagy after SCI

**Figure 4:**
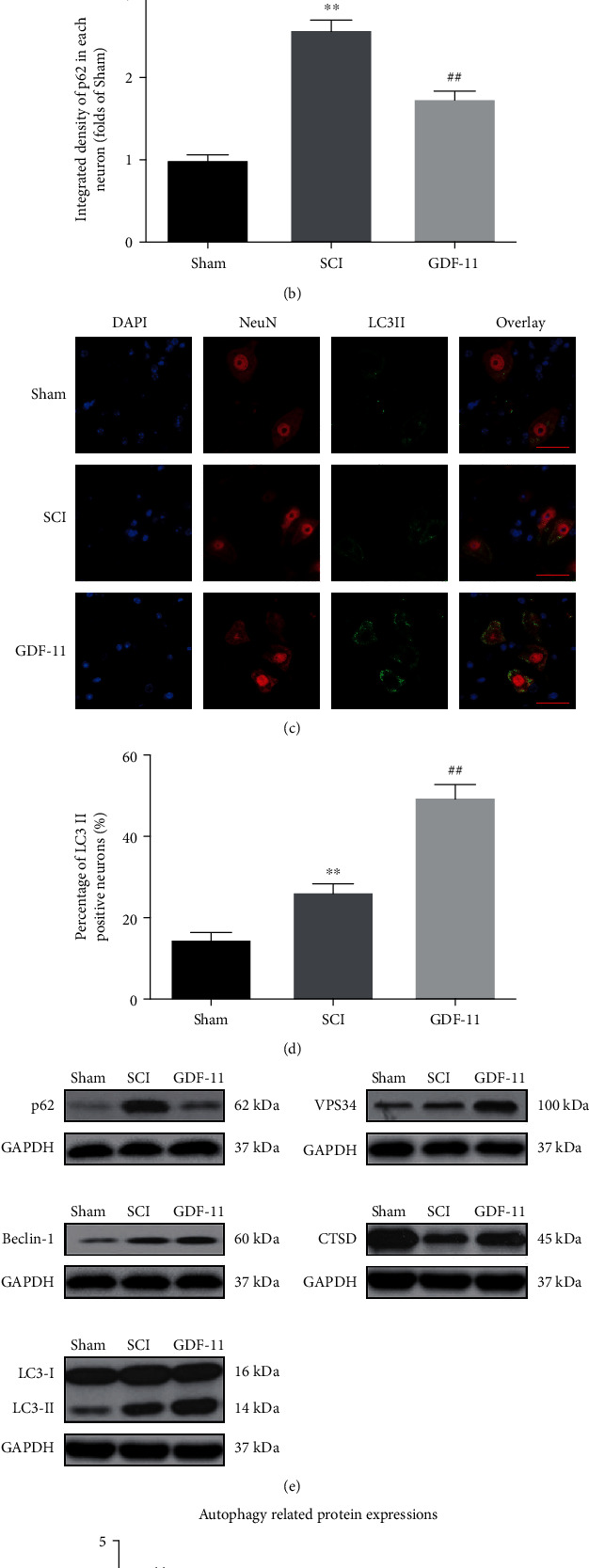
GDF-11 enhances autophagy following spinal cord injury. (a) Immunofluorescence staining for p62 and NeuN colocalization at the spinal cord lesion following spinal cord injury (scale bar = 25 *μ*m). (b) The quantitative mean optical density of p62 in neurons of spinal cord lesions in the respective groups. (c) Staining based on immunofluorescence in terms of the colocalization of NeuN and LC3II within spinal cord lesions following spinal cord injury (scale bar = 25 *μ*m). (d) The percentage of LC3II-positive neurons in neurons of spinal cord lesions in the respective groups. (e) Western blot assay for CTSD, VPS34, Beclin1, LC3II, and p62 expression levels in the sham, SCI, and GDF-11 groups. Gels were subjected to identical experimental conditions, with cropped blots presented. (f) Optical densities of CTSD, VPS34, Beclin1, LC3II, and p62 expression levels were quantified and investigated in the respective groups. Data are expressed as the mean ± SEM, *n* = 6 per group. ^∗^*p* < 0.05 and ^∗∗^*p* < 0.01 vs. the sham group. ^##^*p* < 0.01 vs. the SCI group.

For the evaluation of autophagic activity within spinal cord lesions following SCI, we determined the expression profiles of the autophagic substrate protein (p62), an autolysosome-related marker (CTSD), and autophagosomal markers (Vps34, Beclin1, and LC3II). As shown in Figure 4(a), IF staining revealed that p62 was expressed within neurons in the lesions. According to the quantitative investigation, following SCI, the p62 density in neurons increased markedly (*p* < 0.01); however, the GDF-11 group achieved a relatively low p62 density in neurons in contrast to the SCI group (*p* < 0.01; Figure 4(b)). As shown in Figure 4(c), the spinal cord showed a greater percentage of LC3II-positive neurons in the SCI group than in the sham group (*p* < 0.01); compared with that of the SCI group, GDF-11 treatment upregulated LC3II-positive neurons (*p* < 0.01; Figure 3(d)). WB was performed to ascertain the amounts of CTSD, VPS34, Beclin1, LC3II, and p62 (Figure 3(e)). As revealed by the results, compared with that within the sham group, VPS34, Beclin1, LC3II, and p62 had a higher OD within the SCI group (*p* = 0.02, <0.01, <0.01, and <0.01, respectively), with smaller OD values for CTSD within the SCI group (*p* < 0.01). Compared with the SCI group, the GDF-11 group showed increases in CTSD, VPS34, Beclin1, and LC3II levels but decreases in p62 levels (*p* < 0.01 for all; Figure 3(f)). These results summarize the phenomenon of autophagy substrate accumulation that occurs following SCI, despite the upregulation of autophagosome- and autophagolysosomal-related markers. In addition, as revealed by the abovementioned results, GDF-11 could upregulate autolysosome- and autophagosome-associated markers and alleviate autophagy substrate pressure, which is likely to result from a complete improvement in autophagic flux following SCI.

### 3.5. Suppression of Autophagy Reverses the Influence Exerted by GDF-11 on Pyroptosis and Necroptosis after SCI

**Figure 5:**
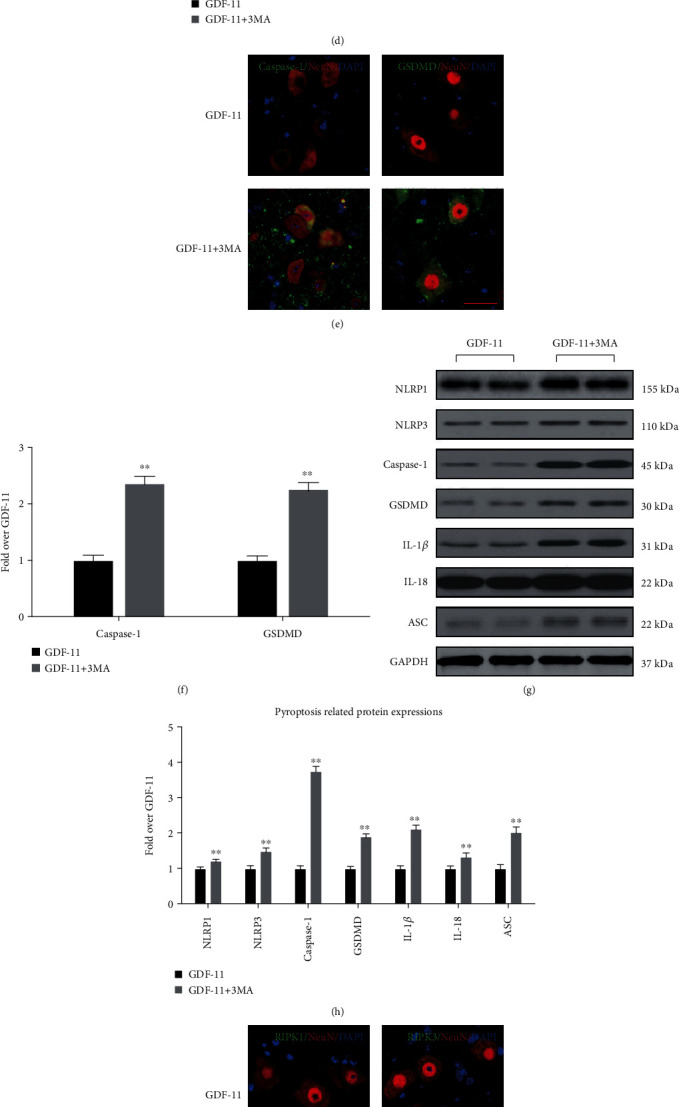
Suppression of autophagy reverses the influence exerted by GDF-11 on pyroptosis and necroptosis following spinal cord injury. (a) Neuron colocalization and immunofluorescence staining for p62 and LC3II at the spinal cord lesion following spinal cord injury (scale bar = 25 *μ*m). (b) The quantitative mean optical density of p62 and the number of LC3II-positive neurons in neurons of spinal cord lesions in the respective groups. (c) Western blot assay for CTSD, VPS34, Beclin1, LC3II, and p62 expression levels in the respective groups. (d) Optical densities of CTSD, VPS34, Beclin1, LC3II, and p62 expression levels were quantified and investigated in the respective groups. (e) Neuron colocalization and immunofluorescence staining for Caspase-1 and GSDMD in the spinal cords of each group (scale bar = 25 *μ*m). (f) The quantitative mean optical density of Caspase-1 and GSDMD in neurons of spinal cord lesions. (g) Western blot assay for IL-18, IL-1*β*, GSDMD, Caspase-1, ASC, NLRP3, and NLRP1 expression levels in the respective groups. (h) Optical densities of IL-18, IL-1*β*, GSDMD, Caspase-1, ASC, NLRP3, and NLRP1 expression levels were quantified and investigated in the respective groups. (i) Neuron colocalization and immunofluorescence staining for RIPK1 and RIPK3 in the spinal cords of each group (scale bar = 25 *μ*m). (j) Quantification of the optical density of RIPK1 and RIPK3 in neurons of spinal cord lesions. (k) Western blot assay for Caspase-8, MLKL, RIPK3, and RIPK1 expression levels in the respective groups. (l) Optical densities of Caspase-8, MLKL, RIPK3, and RIPK1 expression levels were quantified and investigated in the respective groups. Data are expressed as the mean ± SEM, *n* = 6 per group. ^∗^*p* < 0.05 and ^∗∗^*p* < 0.01 vs. the GDF-11 group.

The 3MA, an autophagy suppressor, was coadministered with GDF-11 to assess whether GDF-11 had a conducive influence on the results after SCI resulting from the activation of autophagy. Neuron colocalization analysis and IF revealed an upregulation of the p62 density and downregulation of the percentage of neurons positive for LC3II in the GDF − 11 + 3MA group compared with the GDF-11 group (*p* < 0.01 for both; Figures 5(a) and 5(b)). WB was employed to detect the expression levels of CTSD, VPS34, Beclin1, LC3II, and p62 (Figure 5(c)). Compared with the GDF-11 group, the CTSD, VPS34, Beclin1, and LC3II groups had lower OD values within the GDF − 11 + 3MA group (*p* < 0.01, ≤0.01, <0.01, and =0.01, respectively), with larger OD values for p62 within the GDF − 11 + 3MA group (*p* < 0.01; Figure 5(d)). Thus, 3MA significantly suppressed autophagy during coadministration with GDF-11.

For an in-depth verification of autophagy as the main factor allowing GDF-11 to facilitate neuronal function recovery following SCI, we delved into the influences exerted by 3MA on necroptosis and pyroptosis. As shown in the IF analysis, compared with those within the GDF-11 group, the densities of GSDMD and Caspase-1 within neurons exceeded those within the GDF − 11 + 3MA group (*p* < 0.01 for both; Figures 5(e) and 5(f)). The expression levels of pyroptosis-related proteins (IL-18, IL-1*β*, GSDMD, Caspase-1, ASC, NLRP3, and NLRP1) were higher in the GDF − 11 + 3MA group than in the GDF-11 group (*p* < 0.01 for all; Figures 5(g) and 5(h)). IF also showed that the RIPK1 and RIPK3 densities in neurons were markedly increased in the GDF − 11 + 3MA group compared with the GDF-11 group (*p* < 0.01 for both; Figures 5(i) and 5(j)). The OD values for necroptosis-related proteins (MLKL, RIPK3, and RIPK1) were higher in the GDF − 11 + 3MA group than in the GDF-11 group (*p* < 0.01 for all), with a lower OD value for Caspase-8 in the GDF − 11 + 3MA group (*p* < 0.01; Figures 5(k) and 5(l)). These results indicated that coadministration of 3MA with GDF-11 led to a weakening of the reducing effect of GDF-11 on pyroptosis and necroptosis, thereby demonstrating the underlying autophagy-enhancing effects of GDF-11 in the mechanism by which it inhibited pyroptosis and necroptosis.

### 3.6. Autophagy Inhibition Reverses the Neuroprotective Effects of GDF-11 on SCI

**Figure 6:**
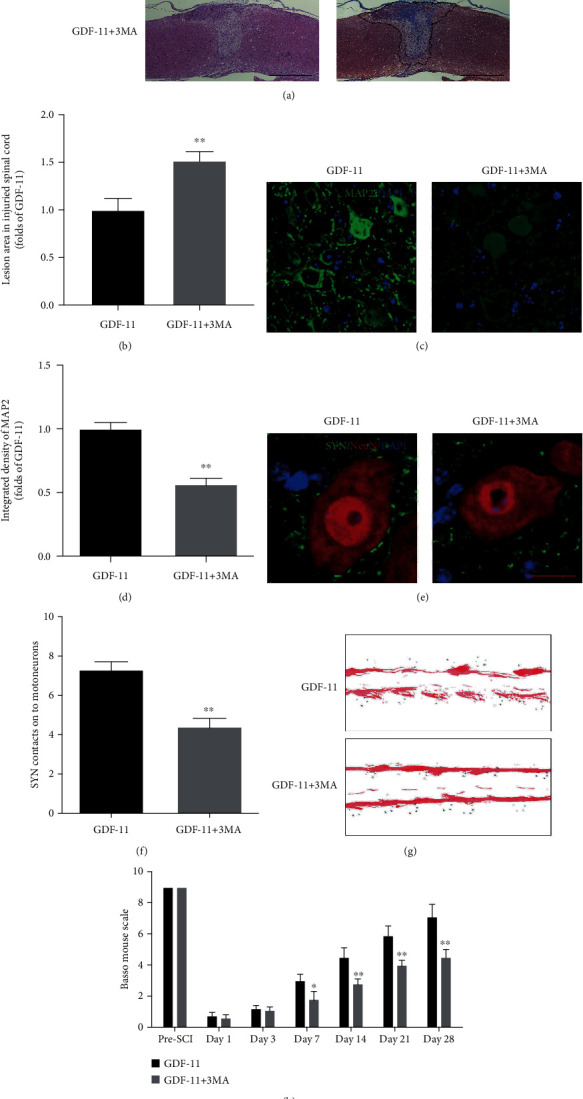
Suppression of autophagy reverses the influence exerted by GDF-11 on functional recovery following spinal cord injury. (a) Longitudinal spinal cord sections in the indicated groups on day 28 were analysed on the basis of Masson staining and HE staining (scale bar = 1000 *μ*m). (b) Quantitative investigation of Masson-positive lesions within the spinal cords of the respective groups. (c) Photographs (×30) of spinal cord sections in the respective groups stained with an antibody against MAP2 (scale bar = 25 *μ*m). (d) MAP2 optical density within spinal cords subjected to injury on day 28. (e) Photographs (×150) of spinal cord sections following injury (T11-T12) and stained on day 28 with an antibody against SYN/NeuN (scale bar = 5 *μ*m). (f) Relevant quantitative results for numbers of neuron-contacting synapses. (g) Photographs of mouse footprints on day 28 following spinal cord injury. (h) Basso mouse scale (BMS) scores for the indicated groups and time points. Data are expressed as the mean ± SEM, *n* = 6 per group. ^∗^*p* < 0.05 and ^∗∗^*p* < 0.01 vs. the GDF-11 group.

The lesion area in the injured spinal cord was assessed by HE and Masson staining, which revealed a broadened area of glial scars (*p* < 0.01, Figures 6(a) and 6(b)) in the GDF − 11 + 3MA group compared with the GDF-11 group. Additionally, IF staining revealed decreased MAP2 states (*p* < 0.01, Figures 6(c) and 6(d)) and a smaller number of SYN-positive synapses on neurons (*p* < 0.01, Figures 6(e) and 6(f)) in the GDF − 11 + 3MA group than in the GDF-11 group. On day 28 following injury, the GDF-11 group displayed a marked restoration of hind leg action, with coordinated crawling, while the GDF − 11 + 3MA group continued to drag their hind legs (Figure 6(g)). An insignificant difference was reported for the BMS score between the GDF-11 and GDF − 11 + 3MA groups on days 1 and 3. In the GDF − 11 + 3MA group, the BMS scores were markedly lower than those in the GDF-11 group after SCI on days 7, 14, 21, and 28 (*p* = 0.04, <0.01, <0.01, and <0.01, respectively; Figure 6(h)). Therefore, the autophagy-improving influence of GDF-11 likely accounted for the optimized results with GDF-11 treatment following SCI.

### 3.7. GDF-11 Facilitates Autophagy by Upregulating TFE3 Activity and Subsequently Depresses Pyroptosis and Necroptosis following SCI

**Figure 7:**
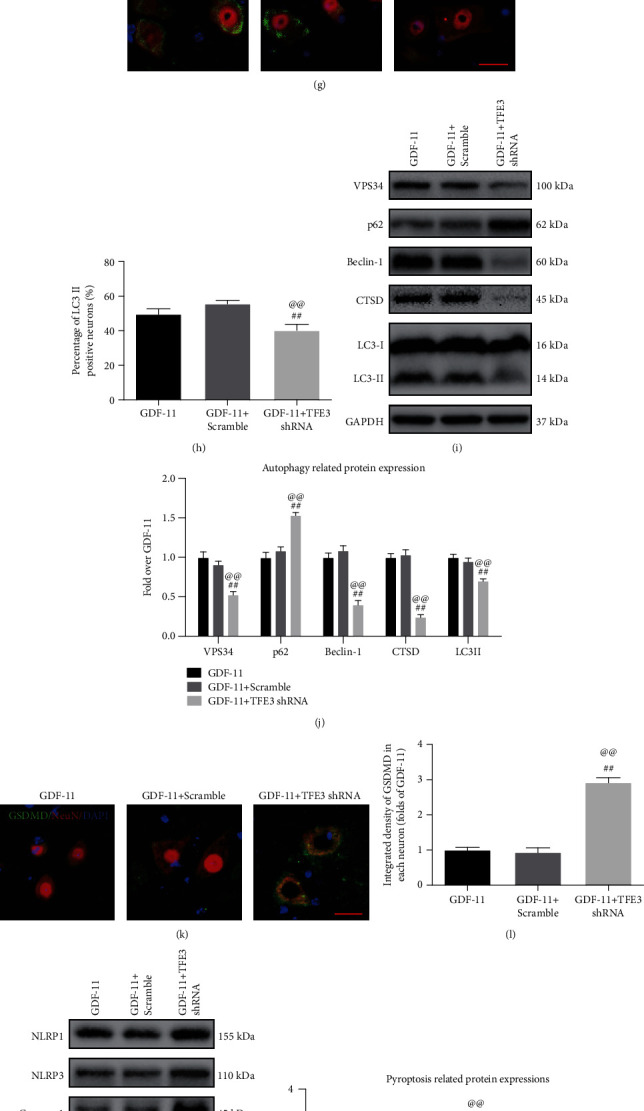
GDF-11 enhances autophagy by upregulating TFE3 activity and inhibiting pyroptosis and necroptosis. (a) Immunofluorescence to detect TFE3 within spinal cord lesions (scale bar = 25 *μ*m). (b) Quantification of the optical density of TFE3 expression in neurons. (c) Western blot assay of nuclear TFE3 and cytoplasmic TFE3 expression. (d) Quantification results for optical densities pertaining to nuclear TFE3 and cytoplasmic TFE3. (e) Western blot assay reporting nuclear TFE3 and cytoplasmic TFE3 expression levels in the GDF-11, GDF − 11 + scramble and GDF − 11 + TFE3 shRNA groups. (f) Quantification results for optical densities pertaining to nuclear TFE3 and cytoplasmic TFE3. (g) Staining based on immunofluorescence in terms of the colocalization of NeuN and LC3II within spinal cord lesions (scale bar = 25 *μ*m). (h) Percentage of LC3II-positive neurons in spinal cord lesions in the respective groups. (i) Western blot assay for CTSD, VPS34, Beclin1, LC3II, and p62 expression levels in the GDF-11, GDF − 11 + scramble, and GDF − 11 + TFE3 shRNA groups. (j) Optical densities of CTSD, VPS34, Beclin1, LC3II, and p62 expression levels were quantified and investigated in the respective groups. (k) Immunofluorescence staining for GSDMD and NeuN colocalization in spinal cord lesions (scale bar = 25 *μ*m). (l) Quantitative average optical density of GSDMD within neurons of spinal cord lesions. (m) Western blot assay for IL-18, IL-1*β*, GSDMD, Caspase-1, ASC, NLRP3, and NLRP1 expression levels in each group. (n) Optical densities of IL-18, IL-1*β*, GSDMD, Caspase-1, ASC, NLRP3, and NLRP1 expression levels were quantified and investigated in the respective groups. (o) Immunofluorescence staining for RIPK1 and NeuN colocalization in spinal cord lesions (scale bar = 25 *μ*m). (p) Quantification of the optical density of RIPK1 in neurons of spinal cord lesions. (q) Western blot assay for Caspase-8, MLKL, RIPK3, and RIPK1 expression levels in the three groups. (r) Optical densities of Caspase-8, MLKL, RIPK3, and RIPK1 expression levels were quantified and investigated in the respective groups. Data are expressed as the mean ± SEM, *n* = 6 per group. ^∗^*p* < 0.05 and ^∗∗^*p* < 0.01 vs. the SCI group. ^#^*p* < 0.05 and ^##^*p* < 0.01 vs. the GDF-11 group. ^@^*p* < 0.05 and ^@@^*p* < 0.01 vs. the GDF − 11 + scramble group.

We then explored TFE3 expression in the cytoplasm and nucleus to determine whether GDF-11 had a regulatory effect on TFE3. As shown in Figures 7(a) and 7(b), after GDF-11 treatment, TFE3 expression in neurons was markedly increased (*p* < 0.01). Next, according to the WB results, intranuclear TFE3 expression rose markedly in the GDF-11 group, while TFE3 expression in the cytoplasm was decreased (*p* < 0.01 for both; Figures 7(c) and 7(d)). For an in-depth confirmation of the effect of TFE3 activation in promoting autophagy and inhibiting pyroptosis and necroptosis through GDF-11, we silenced the TFE3 activity using TFE3 shRNA and designed a trial to compare the following three groups: GDF-11 only, GDF − 11 + scrambled shRNA, and GDF − 11 + TFE3 shRNA. The results showed that both cytoplasmic TFE3 expression and nuclear TFE3 expression in the TFE3 shRNA group were markedly lower than those in the scramble group, whereas an insignificant difference was reported for the nuclear TFE3 expression status between the GDF-11 and GDF − 11 + scramble groups (*p* < 0.01 for all; Figures 7(e) and 7(f)). These results indicated that TFE3 shRNA transfection suppressed TFE3 expression and nuclear translocation.

**Figure 8:**
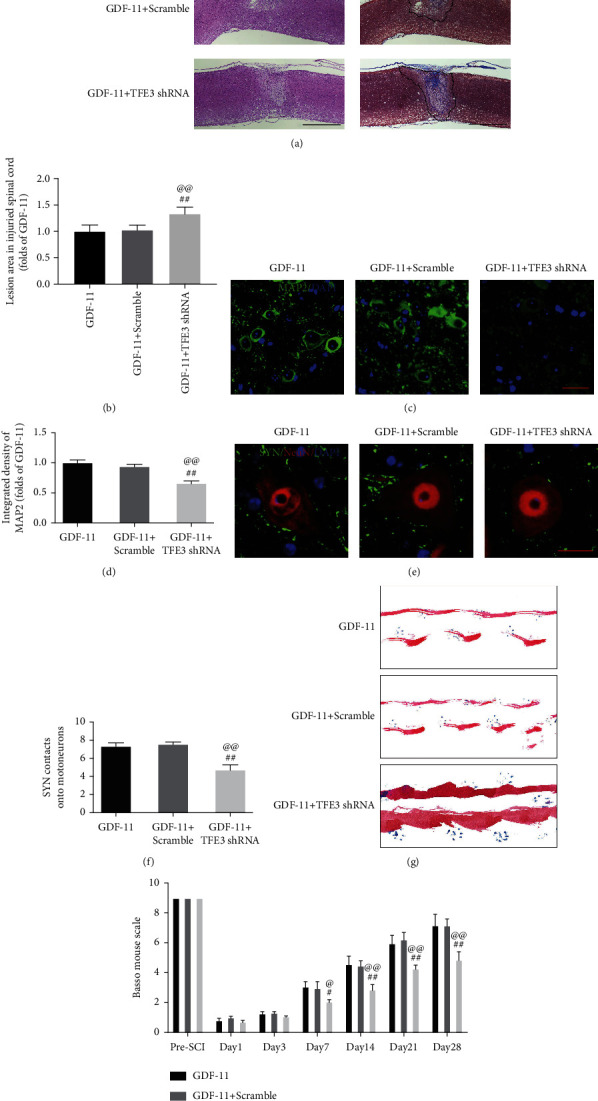
Autophagy inhibition reverses the neuroprotective effects of GDF-11 on SCI. (a) Longitudinal spinal cord sections in the indicated groups on day 28 were analysed on the basis of Masson staining and HE staining (scale bar = 1000 *μ*m). (b) Quantitative investigation of Masson-positive lesions within the spinal cords of the respective groups. (c) Photographs (×30) of spinal cord sections in the respective groups stained using an antibody against MAP2 (scale bar = 25 *μ*m). (d) MAP2 optical density within spinal cords subjected to injury on day 28. (e) Photographs (×150) of spinal cord sections following injury (T11-T12) and stained on day 28 with an antibody against SYN/NeuN (scale bar = 5 *μ*m). (f) Relevant quantification results for numbers of neuron-contacting synapses. (g) Photographs of mouse footprints on day 28 following spinal cord injury. (h) Basso mouse scale (BMS) scores in terms of the indicated groups and time points. Data are expressed as the mean ± SEM, *n* = 6 per group. ^#^*p* < 0.05 and ^##^*p* < 0.01 vs. the GDF-11 group. ^@^*P* < 0.05 and ^@@^*P* < 0.01 vs. the GDF − 11 + scramble group.

Subsequently, we conducted studies to determine whether TFE3 nuclear translocation induced by GDF-11 was responsible for the regulation of autophagy, pyroptosis, and necroptosis. IF revealed an insignificant distinction in the percentage of neurons positive for LC3II between the GDF-11 and GDF − 11 + scramble groups, while the proportion in the GDF − 11 + TFE3 shRNA group was markedly reduced (*p* < 0.01 for both; Figures 7(g) and 7(h)). Likewise, according to the WB results, the expression levels of p62, LC3II, CTSD, VPS34, and Beclin1 were not markedly different between the GDF-11 and GDF − 11 + scramble groups and the expression levels of VPS34, Beclin1, CTSD, and LC3II were markedly lower in the GDF − 11 + TFE3 shRNA group than in the GDF − 11 + scramble group, while opposite results were obtained for p62 (*p* < 0.01 for all; Figures 7(i) and 7(j)). TFE3 shRNA treatment increased the expression levels of pyroptosis-associated markers (ASC, IL-18, IL-1*β*, GSDMD, Caspase-1, NLRP3, and NLRP1) and necroptosis-related markers (RIPK1, RIPK3, MLKL, and opposite Caspase-8), as shown in Figures 7(k)–7(r) (*p* < 0.01 for all). The GDF − 11 + TFE3 shRNA group showed a broadened area of glial scarring (*p* = 0.01, Figures 8(a) and 8(b)), decreased MAP2 expression (*p* < 0.01, Figures 8(c) and 8(d)), and fewer SYN-positive synapses (*p* < 0.01, Figures 8(e) and 8(f)) on neurons compared with the GDF-11 group following SCI. On day 28 following injury, the GDF − 11 + TFE3 shRNA group was still dragging their hind legs (Figure 8(g)). An insignificant difference was reported for the BMS score among the GDF-11, the GDF − 11 + scramble, and GDF − 11 + TFE3 shRNA groups on days 1 and 3. In the GDF − 11 + TFE3 shRNA group, the BMS scores were markedly lower than those in the GDF-11 and GDF − 11 + scramble groups on days 7, 14, 21, and 28 after SCI (*p* = 0.03, <0.01, <0.01, and <0.01; Figure 8(h)). Together, these results suggested that TFE3 activation and nuclear translocation were the major mechanisms by which GDF-11 increased autophagy and inhibited pyroptosis and necroptosis.

### 3.8. GDF-11 Activates TFE3 through the AMPK-TRPML1-Calcineurin Signalling Pathway after SCI

**Figure 9:**
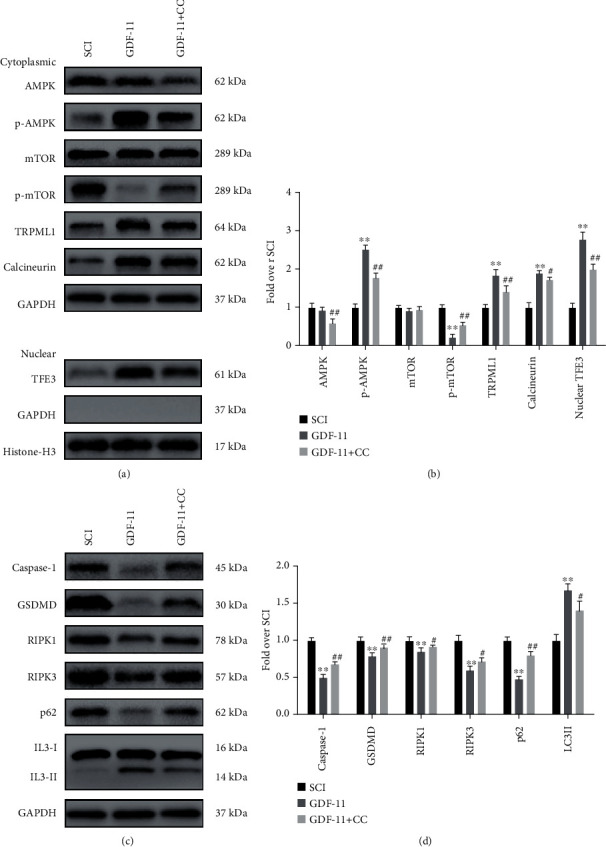
GDF-11 activates TFE3 through the AMPK-TRPML1-calcineurin signalling pathway. (a) Western blot assay showing the cytoplasmic expression levels of p-mTOR, mTOR, p-AMPK, AMPK, TRPML1, and calcineurin, normalized to GAPDH as an internal control; nuclear expression of TFE3, normalized to histone H3 as an internal control. (b) Optical densities of TRPML1, p-mTOR, mTOR, p-AMPK, AMPK, calcineurin, and nuclear TFE3. (c) Western blot assay showing the expression levels of Caspase-1, GSDMD, RIPK1, RIPK3, p62, and LC3II normalized to GAPDH as an internal control. (d) Optical densities of Caspase-1, GSDMD, RIPK1, RIPK3, p62, and LC3II. Data are expressed as the mean ± SEM, *n* = 6 per group. ^∗^*p* < 0.05 and ^∗∗^*p* < 0.01 vs. the SCI group. ^#^*p* < 0.05 and ^##^*p* < 0.01 vs. the GDF-11 group.

According to published reports, there is an important calcium signalling pathway that modulates TFE3: the AMPK-TRPML1-calcineurin signalling cascade. Our results showed that GDF-11 increased p-AMPK expression and TFE3 nuclear translocation, while p-mTOR was inhibited (*p* < 0.01 for all, Figures 9(a) and 9(b)). As downstream signalling molecules, Western blot analysis revealed that the expression states of TRPML1 and calcineurin were markedly increased (*p* < 0.01 for both, Figures 9(a) and 9(b)). To further investigate whether TFE3 activation after GDF-11 treatment was modulated by the AMPK-TRPML1-calcineurin signalling pathway, we explored the effects of compound C (CC), an AMPK blocker, on the GDF-11 group. Here, p-AMPK expression and TFE3 nuclear translocation states were lower in the GDF − 11 + CC group than in the GDF-11 group (*p* < 0.01 for both), while p-mTOR expression was higher in the GDF − 11 + CC group (*p* < 0.01, Figures 9(a) and 9(b)). Likewise, the expression levels of TRPML1 and calcineurin were markedly decreased (*p* < 0.01 and *p* = 0.04, respectively, Figures 9(a) and 9(b)). We further evaluated whether the AMPK-TRPML1-calcineurin axis was also involved in the mechanism by which GDF-11 modulated pyroptosis-, necroptosis-, and autophagy-related proteins. The WB results showed that the expression levels of Caspase-1, GSDMD, RIPK1, RIPK3, and p62 were markedly higher in the GDF − 11 + CC group than in the GDF-11 group, while LC3II expression levels showed the opposite pattern (*p* < 0.01, <0.01, =0.04, =0.03, <0.01, and =0.01, Figures 9(c) and 9(d)). Together, our results confirmed that GDF-11 activated TFE3 through the AMPK-TRPML1-calcineurin signalling cascade.

## 4. Discussion

Significant damage to the spinal cord is capable of causing sensorimotor disorder or permanent paralysis [49]. A decreased regenerative capacity (neuronal death) after SCI is capable of disrupting the continuing signal transmission property within the limbs and brain, as well as hindering functional recovery [50, 51]. Thus, it is essential to inhibit neuronal death and facilitate neuronal regeneration, which is capable of forming novel relay circuits to replace ruptured circuits. GDF-11 is an important regulator of central nervous system formation and fate throughout life [52]. In previous studies, GDF-11 exerted neuroprotective and neurorestorative effects on cerebral ischaemic injury [39, 53]. Programmed cell death, including the pyroptosis, necroptosis, and autophagy, is found to facilitate neurological deficits [54–57]. Thus, we hypothesized that GDF-11 regulates pyroptosis, necroptosis, and autophagy following SCI. In this study, our results showed that the therapeutic effect of GDF-11 was due to the activity of TFE3 through the AMPK-TRPML1-calcineurin signalling pathway, which subsequently enhanced autophagy and further attenuated pyroptosis and inhibited necroptosis.

Pyroptosis refers to one proinflammatory form of programmed cell death that is regulated during the process of activating caspase-1-, caspase-4/5/11-, and GSDMD-regulated signalling pathways and the release of several inflammatory mediators, such as IL-1*β* and IL-18 [58–61]. By assembling inflammasome-related sensor proteins (NLRP1, NLRP3, AIM2, and pyrin), the scaffolding protein apoptosis-related speck-like protein covering CARD (ASC), and proinflammatory caspase (caspase-1 and -4/5 in humans) into inflammasomes, the autoactivating process for caspase and subsequent proteolytic gasdermin D (GSDMD) cleavage can be promoted, thereby triggering cell pyroptosis [12]. To evaluate GDF-11 activity in pyroptosis, we performed IF staining to assess the Caspase-1 and GSDMD density in neurons and found diminished GDF-11 densities in spinal cord lesions. According to the WB results, following GDF-11 treatment, IL-1B, IL-1*β*, Caspase-1, GSDMD, NLRP1, NLRP3, and ASC expression levels were all decreased, thereby demonstrating that GDF-11 was an effective suppressor of pyroptosis in the mouse SCI model.

Necroptosis is regulated by classical necrosomes that comprise mixed-lineage kinase domain-like protein (MLKL) and receptor-interacting protein kinase 1/3 (RIPK1/3) via TNF/TNFR1 signalling or other stimuli [18]. RIPK1, an upstream mediator of necrosis, is a key regulator of innate immune responses involved in inflammation and cell death [33]. RIPK3 plays a pivotal role in the recruitment and phosphorylation of MLKL, which is the executor of necroptosis to recruit Ca^2+^ and Na^+^ ion pathways and form pores at the plasma membrane, leading to cell rupture [62]. Therefore, they may be ideal targets for reducing cell death and inflammation in the central nervous system [63]. Thus, we hypothesized that GDF-11 might inhibit necroptosis following SCI. In this study, we showed via both IF and WB that GDF-11 decreased expression levels of MLKL, RIPK3, and RIPK1, while Caspase-8 expression was increased. Therefore, our results demonstrated that GDF-11 could inhibit necroptosis in SCI.

Autophagy, a major process of degrading intracellular waste, has an important role in maintaining cellular homeostasis [64]. After SCI, lysosomal damage and dysfunction can occur within affected cells, leading to autophagy flux defects, an accumulation of autophagosomes, and active cell death, which is not conducive to the survival of neurons [65]. A previous study has demonstrated that autophagy is suppressed by knocking out the ATG5 gene, further demonstrating that functional recovery can be limited by blocking autophagy after SCI [66]. Here, from the results of increased expressions of Beclin1, VPS34, CTSD, LC3II, and decreased p62 expression via both IF and WB, we revealed that GDF-11 functioned as an effective activator of autophagy by improving the states of autophagy flux.

Neuronal death via pyroptosis or necroptosis is regulated through the activities of host proteins that induce different biological outcomes [67]. Autophagy, a prosurvival mechanism, has been found to suppress pyroptosis and necroptosis by degradation of various proteins (e.g., NLRP3, ASC, and RIPK1) [35, 36, 68]. Previous studies of SCI have confirmed that the induction of autophagy plays a neuroprotective role by suppressing apoptosis [69]. However, few studies investigated the effect of autophagy on cosuppression of pyroptosis and necroptosis following SCI. In the current study, we used 3MA to inhibit autophagy in SCI with GDF-11 treatment to demonstrate that GDF-11 inhibited pyroptosis and necroptosis via autophagy enhancement. After autophagy was suppressed, pyroptosis-associated and necroptosis-associated markers were partially adjusted and functional recovery was inhibited in SCI with GDF-11 treatment. However, the specific connection and biochemical mechanism need to be further studied. Altogether, these results suggest that GDF-11 suppresses pyroptosis and necroptosis by activating autophagy, which plays a critical role in the therapeutic effect of GDF-11 after SCI.

To elucidate the GDF-11 action system and how it facilitates autophagy in SCI, we also explored upstream mechanisms of autophagy activity. Previous reports have shown that autophagy is regulated by TFE3, which is a member of the MiT/TFE subfamily of the bHLH-LZ transcription factor family [70, 71]. Therefore, in this study, we investigated the relationship between tissue TFE3 and autophagy activity. From a mechanistic perspective, within a normal physiological environment, phosphorylated TFE3 interacts with 14–3–3 cytosolic chaperones to form the TFE3-14-3-3 complex; if cells receive stimulation from environmental signals (starvation, hypoxia, or toxins), TFE3 dephosphorylation inhibits the activity of mTOR and causes it to separate from the TFE3-14-3-3 complex [72]. In our study, we showed that GDF-11 increased TFE3 expression. Simultaneously, activated TFE3 markedly enhanced autophagy, attenuated pyroptosis, inhibited necroptosis, and promoted functional recovery in the SCI group. Taken together, these results demonstrated that the therapeutic effect of GDF-11 was regulated by promoting the nuclear translocation of TFE3.

Next, we also investigated how GDF-11 regulated TFE3 states. AMPK, which is capable of responding to a low-energy state and starvation, can be activated by the ratio of AMP/ATP, Ca^2+^-activated Ca^2+^/calmodulin-dependent kinase (CaMKII), and converting growth factor-*β*-activated kinase 1 (TAK1) [73]. Activation of the AMPK-mTOR pathway also facilitates the release of Ca^2+^ via the TRPML1 pathway, thereby activating calcineurin [74, 75]. In the past few years, it has been reported that the PPP3/calcineurin-activating process and increased intracellular Ca^2+^ states may stimulate TFE3 dephosphorylation and the translocation of TFE3 to the nucleus following SCI [76, 77]. Here, our results suggested that the AMPK-TRPML1-calcineurin signalling pathway was activated after GDF-11 treatment in SCI. With the use of CC, suppressing the AMPK-TRPML1-calcineurin signalling pathway could reduce the influence exerted by GDF-11 on autophagy, pyroptosis, and necroptosis.

This work has several limitations that will require in-depth analyses. Specifically, in a previous study, TFE3 activation during SCI was found to be partially regulated by AMPK-SKP2-CARM1 and AMPK-mTOR signalling pathways [2, 68]. In-depth investigations can delve into whether GDF-11 also acts through AMPK-SKP2-CARM1 and AMPK-mTOR signalling pathways and the differences among them in SCI. The expression of GDF-11 decreases gradually with age, and more work should be performed to investigate whether the recovery of patients of different ages depends on the expression of GDF-11. The optimal dose and regimen of GDF-11 will require in-depth assessments to improve its therapeutic value.

We also have several prospects for further research on GDF-11. A previous study has demonstrated that TFE3 and TFEB share partial common mechanisms [70]. Therefore, we speculate that GDF-11 is also likely to activate autophagy through TFEB enhancement after SCI but the details require further study. It was found that the treatment of GDF-11 for central nervous system injury is promising [53, 78]. However, there is a lack of clinical experimental reports related to GDF-11 in CNS injury. Therefore, the possibility of clinical transformation of GDF-11 for SCI requires further research. Many other mechanisms may also cause neuronal death following SCI, such as parthanatos. Parthanatos have a close relationship with nervous system diseases and neurologic disorders and have been described in nervous system neoplasms [79]. The effect of GDF-11 on parthanatos after SCI is an issue worthy of further discussion.

In conclusion, our study demonstrated that GDF-11 facilitated the nuclear translocation of TFE3 by activating the AMPK-TRPML1-calcineurin signalling pathway, which enhanced autophagy. Subsequently, increased autophagy attenuated pyroptosis and inhibited necroptosis. Ultimately, the effects of GDF-11 that were evaluated facilitate functional recovery following SCI. In conclusion, we not only focused on the effect of GDF-11 on autophagy, pyroptosis, and necroptosis but also demonstrated the connection among them. The results of our studies show that replenishment with GDF-11 may be a novel therapeutic approach with broad clinical potential for the treatment of SCI patients.

## Data Availability

The datasets used and analysed during the current study are available from the corresponding authors upon reasonable request.
